# Novel mutations of *TEX11* are associated with non-obstructive azoospermia

**DOI:** 10.3389/fendo.2023.1159723

**Published:** 2023-04-14

**Authors:** Jian Song, Yanwei Sha, Xiaojun Liu, Xuhui Zeng, Xiuling Zhao

**Affiliations:** ^1^ Affiliated Hospital of Nantong University, School of Medicine, Nantong University, Nantong, China; ^2^ School of Medicine, Nantong University, Nantong, China; ^3^ Department of Andrology, Women and Children’s Hospital, School of Medicine, Xiamen University, Xiamen, Fujian, China; ^4^ Fujian Provincial Key Laboratory of Reproductive Health Research, School of Medicine, Xiamen University, Xiamen, Fujian, China; ^5^ State Key Laboratory of Molecular Vaccinology and Molecular Diagnostics, School of Public Health, Xiamen University, Xiamen, Fujian, China

**Keywords:** azoospermia, TEX11 mutation, meiosis, infertility, WES

## Abstract

Non-obstructive azoospermia (NOA) affects 10% of infertile men worldwide, and genetic studies revealed that there are plenty of monogenic mutations that responsible for a part of idiopathic NOA cases. Testis-expressed gene 11 (*TEX11*) is an X-linked meiosis-specific gene, many pathogenic variants in *TEX11* have been detected in NOA patients, and the deficiency of this gene can cause abnormal meiotic recombination and chromosomal synapsis. However, many NOA-affected cases caused by *TEX11* mutation remain largely unknown. This study reported three novel *TEX11* mutations (exon 5, c.313C>T: p.R105*), (exon 7, c.427A>C: p.K143Q) and (exon 29, c.2575G>A: p.G859R). Mutations were screened using whole-exome sequencing (WES) and further verified by amplifying and sequencing the specific exon. Histological analysis of testicular biopsy specimens revealed a thicker basement membrane of the seminiferous tubules and poorly developed spermatocytes, and no post-meiotic round spermatids or mature spermatozoa were observed in the seminiferous tubules of patients with *TEX11* mutation.

**Conclusion:**

This study presents three novel variants of *TEX11* as potential infertility alleles that have not been previously reported. It expanded the variant spectrum of patients with NOA, which also emphasizes the necessity of this gene screening for the clinical auxiliary diagnosis of patients with azoospermia.

## Introduction

Infertility affects approximately 15% of couples worldwide, males accounts for half to infertility (1). Non-obstructive azoospermia (NOA) is defined by absence of spermatozoa in the seminal fluid, and 80% of male infertility with NOA were thought to be idiopathic (2–4). Genetic testing is an important tool in the diagnosis of severe male infertility due of the high prevalence of genetic abnormalities in these patients (5). Numerous attempts have been made to link the gene mutations and azoospermia, the genetic basis of NOA is still unknown in the majority of infertile men. New technological advances for genetic diagnosis has enabled a substantial increase in our understanding about the etiology of male infertility. Research in the mutations involved in male infertility will help us to identify potential molecular targets for contraception, it can also improve genetic counseling for infertility patients seeking for effective treatments in humans.

Meiosis is a specialized cell division program, homologous chromosomes undergo pairing, synapsis, recombination, and faithful segregation in the process (6). Defects in meiosis is one of the important etiologies of infertility and birth defects in humans (6). Although numerous genes involved in meiosis have been specifically identified in the regulation of fertility (7–11), efforts to discover single-gene mutations that contribute to human spermatogenic failure have been mostly unavailing. As an X-chromosome encoded meiosis-specific protein, TEX11 was reported to be present in late-pachytene spermatocytes and in round and elongated spermatids (4), and the high expression of TEX11 in spermatogonia and spermatocytes indicates a critical role of TEX11 in the early stage of germ cell development. Extensive classic experiments using mice models have contributed significantly to how we understand the role of TEX11 in chromosomal synapsis and meiotic recombination (12, 13). TEX11 was proved to be an important component of meiotic nodules needed for recombination, and in *TEX11* mutant mice, spermatogenesis is impaired due to delayed repair of double-strand breaks (DSB) and decreased crossover formation in spermatocytes (14). More specifically, TEX11 may provide a physical link between chromosomal synapsis and meiotic recombination by interacting with SYCP2 *in vivo*, which is an indispensable component of the synaptonemal complex lateral elements, and defects in TEX11 caused apoptosis of spermatocytes at the pachytene stage and male infertility (6).

The homology of amino acid sequences in human and mouse indicates the similarity of function in spermatogenesis. Recently, X-linked *TEX11* mutations have been identified in azoospermic men (3, 4). Yatsenko et al. identified six different *TEX11* mutations, including a deletion mutation of 79 amino acids within the meiosis-specific sporulation domain SPO22, three splicing mutations and two missense mutations, theses mutations were occurred in 2.4% of men with azoospermia and 15% of azoospermia patients with meiotic arrest (4). Yang et al. reported 18 singleton variants in azoospermic men, which included a frameshift mutation, five missense mutations, two silent mutations and the remaining 10 were intronic mutations in *TEX11*. Specifically, the incidence of mutation in men with spermatogenic failure is higher than in controls (7.3% vs 1.7%) (3). Moreover, another recent study of *TEX11* mutations in patient with NOA, they identified seven potential pathogenic mutations, and 1.5% of the 479 patients with NOA carried *TEX11* mutations (15). Given the high incidence of *TEX11* mutations, this gene could be a significant candidate for the clinical evaluation of azoospermia.

In the present study, we reported three novel *TEX11* mutations in the patients with severe non-obstructive azoospermia and analyzed the genetic causes by WES. In addition, we summarized the mutations of *TEX11* related to male infertility.

## Methods and results

### Subjects

Pedigrees of the three families were recruited from the Reproductive Medicine Center of the Maternal and Child Care Hospital of Xiamen. Proband semen analysis was performed according to the guidelines of the World Health Organization 2010 guidelines for patients, who were diagnosed with NOA and confirmed using testicular fine needle aspiration.

### Ethical approval

All procedures involving human participants were performed in accordance with the ethical standards of the Ethics Committee of the Maternal and Child Care Hospital of Xiamen. Written informed consents were obtained from all participants.

### Karyotype and AZF deletion analysis

Karyotype analysis was carried out as described previously, peripheral blood lymphocytes (PBL) were collected to confirm the chromosomal status and cytogenetic chromosomal karyotype. PBL were treated with 20 mg/mL colcemid for 1 h to stay at metaphase. G-banding of metaphase chromosomes was performed by Giemsa staining. A total of 20-100 metaphase cells were counted and described by the G-banding method according to the international system for chromosome nomenclature. According to the result of karyotype analysis, the karyotype of all patients was normal (46, XY), and no gonadal mosaicism was observed. PCR was used to detect Y chromosome microdeletions in azoospermia factor regions (AZFa, b, and c). Genomic DNA (gDNA) was isolated from peripheral blood lymphocytes using a QIAamp Blood Mini Kit (Qiagen, Hilden, Germany). The gDNA was amplified using markers (sY84, sY127, sY255, sY86, sY134, and sY254) to detect AZF microdeletions, *SRY* gene was used as internal quantity control, the primers used for PCR were listed in **Supplementary Table 1**, and no microdeletions were detected at AZF loci in either patient. Moreover, the endocrine hormone levels of patients were normal (**Table 1**). 

**Table 1:** The clinicopathological variables of four infertile patients.

Infertility-related examination of affected individuals				
Study participants	P1	P2	P3	P4
Age at last visit (y)	26	24	32	31
Infertility duration (y)	2.5	1.5	3.5	3
Height at last visit (cm)	170	168	171	166
Weight at last visit (kg)	74	72	76	70
Testicular volume (mL)	10	10	12	10
Hormone levels				
FSH (IU/L)	5.02	6.31	6.12	5.60
LH (IU/L)	5.64	5.23	5.84	5.21
T (ng/mL)	4.06	4.28	5.06	4.68
PRL(ng/mL)	12.15	11.85	13.15	9.15
Genetic investigation Karyotype	46, XY	46, XY	46, XY	46, XY
Y-chromosome microdeletion	No	No	No	No

### Whole-exome sequencing

**Figure 1:**
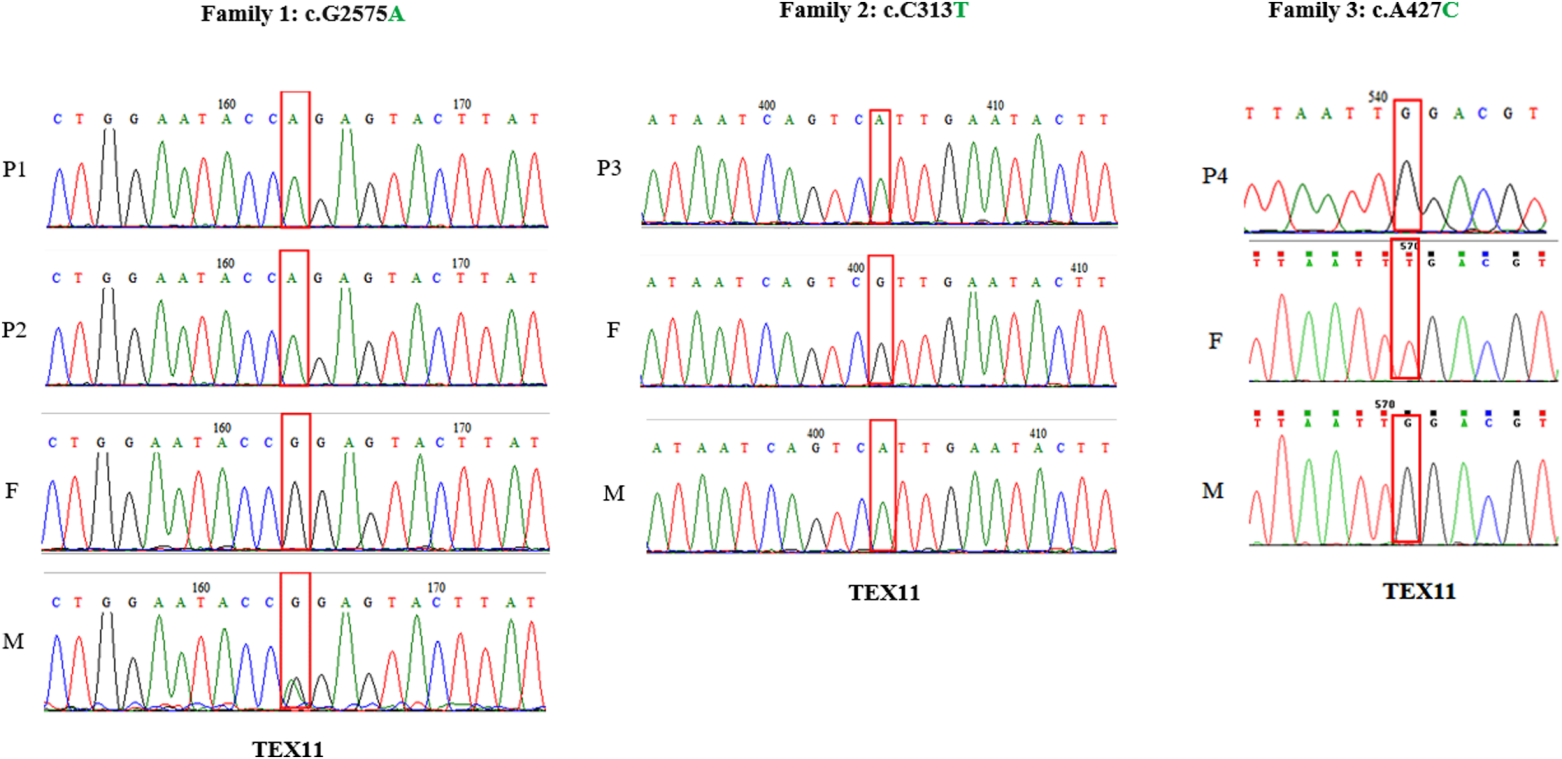
Analysis of the TEX11 variants, the red box indicates the mutation site. Family 1 shows the pedigrees of two brothers (P1, P2) with azoospermia and the inherited TEX11 missense mutation locate in exon 29, c.G2575A; Family 2 shows the pedigrees of P3 inherited TEX11 nonsense mutation locate in exon5, c. C313T; Family 3 shows the pedigrees of P4 inherited TEX11 missense mutation locate in exon7, c.A427C.

**Figure 2:**
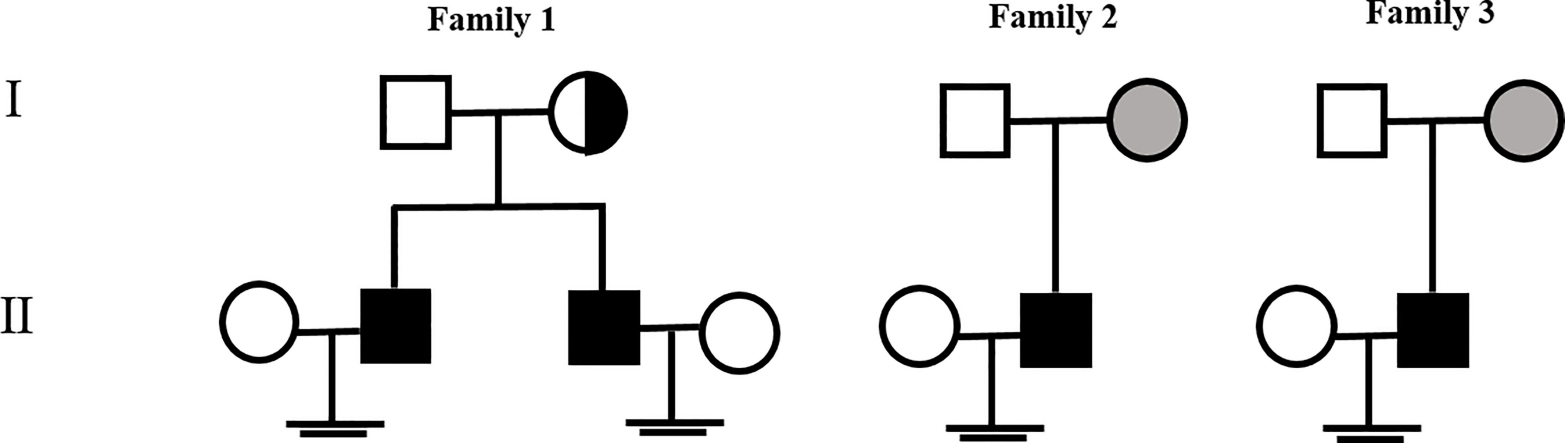
Family pedigree of the proband affected by NOA with a TEX11 mutation. The gray symbols indicate the patient’s mother who is the homozygous carrier of the TEX11 mutation, and the dotted-circle symbols indicate the patient’s mother who is the heterozygous carrier of the TEX11 mutation.

Genomic DNA samples from the three families were extracted from peripheral blood using a QIAamp DNA Blood Midi Kit (Qiagen, Hilden, Germany). WES was performed by Beijing Genome Institute at Shenzhen in the HiSeq2000 sequencing platform (Illumina, San Diego, CA, USA) as described elsewhere (16). Sequencing data were analyzed using Genome Analysis Toolkit Best Practices. (https://software.broadinstitute.org/gatk/best-practices/). Here, we sequenced the whole exome of azoospermia patients with meiotic arrest and found three novel *TEX11* mutations. (exon 29, c.2575G>A) in patient 1, 2 (P1, 2) and (exon 7, c.427A>C) in patient 4 (P4) were missense mutation, and (exon 5, c.313C>T) in patient 3 (P3) was nonsense mutation. Single nucleotide variation of c.427A>C was occurred in 0.0106% of humans according to GnomAD database, and the clinical significance was thought to be benign. And the other two missense mutations were not determined up to now. PCR and Sanger sequencing were used to validate the mutations detected by WES. Primer sequences used for detection of these mutations are shown in **Supplementary Table 2**. In this study, we identified three novel mutations, c.313C>T, c.427A>C and c.2575G>A (**Figure 1**), all of which were inherited from their mother (**Figure 2**), and no pathogenic biallelic or other mutations were found. This suggests that mutations in *TEX11* carried by the proband may underlie their infertility.

### Histological analysis

**Figure 3:**
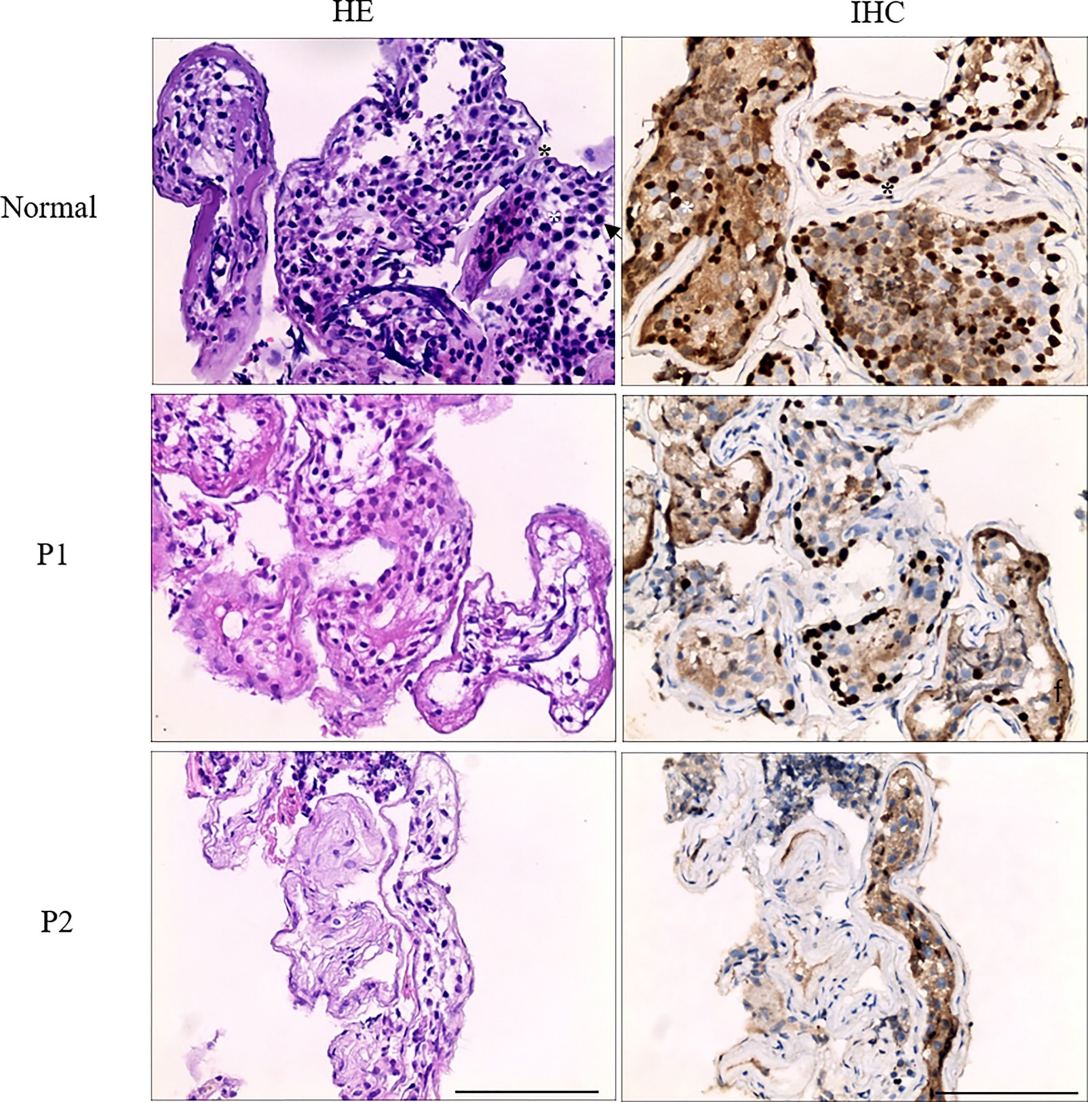
Analysis of testis biopsy samples from family 1 (P1, P2) and normal control. Primary spermatocytes and round spermatids were observed in the normal seminiferous tubules of the testis. Histologic sections showing a thicker basement membrane of the seminiferous tubules and poorly developed spermatocytes, no post-meiotic round spermatids or mature spermatozoa were observed in the seminiferous tubules of patient 1 and patient 2, in contrast with the normal testicular histology. Black asterisk denotes spermatogonia, white asterisk denotes spermatocytes, and arrowhead denotes round spermatids. Scale bar = 100 μm.

To characterize the nature of azoospermia patients, histological examination and TEX11 staining in testicular biopsy were performed. For histology, testicular tissues were obtained by testicular fine needle aspiration from the patient and immediately fixed in Bouin fixative at 4°C overnight, dehydrated in graded ethanol, embedded in paraffin, and cut into 4-μm-thick sections. To examine testicular histology, the sections were deparaffinized in xylene, rehydrated in graded ethanol, and stained with hematoxylin and eosin (H&E), stained sections were examined microscopically. Spermatogenesis was scored according to Johnsen’s scoring system. As for the tubule structure, pathological examination of the patient showed a thicker basement membrane of the seminiferous tubules and poorly developed spermatocytes, no post-meiotic round spermatids or mature spermatozoa. Immunohistochemical staining of TEX11 indicated positive staining spermatogonia, spermatocytes, round spermatids, and mature spermatozoa in the seminiferous tubules of normal testis; TEX11 was detected in spermatogonia and spermatocytes, and absence of staining in post-meiotic round spermatids or mature spermatozoa of mutant seminiferous tubules for the impaired meiosis process in the testicular biopsies (**Figure 3**).

## Discussion and conclusion

Infertility affects a great number of couples worldwide, and male infertility accounts for nearly half of reproductive health problem. The majority of causes of non-obstructive azoospermia in humans are deemed to be idiopathic, and genetic defects are postulated to be the underlying causes. Spermatogenesis is a complex and continuous process controlled by thousands of genes, and any change in the expression or function of these genes may impair the process of spermatogenesis and lead to male infertility (17, 18). It has been reported that genetic variations are probably associated with idiopathic male infertility (19), and identification of stage-specific genes and investigation of novel mutations involved in spermatogenesis are crucial for uncovering the mechanism of male infertility. Sex chromosomes play a key role in germ cell development in men. An increasing number of genes located on the X chromosome have been found to be involved in meiosis. In addition, many X-derived retrogenes such as *Utp14b* (20), *Pgk2* (21), *Cetn1* (22), *Rpl10l* (23), and *Cstf2t* (24) have been confirmed to initiate transcription during male meiosis, and alterations of meiotic proteins results in failure of gametogenesis, which lead to partial or complete sterility. To date, a large amount of singletons had been identified in patients with azoospermia, including exonic missense mutations, exonic frameshift mutations, and intronic mutations.

As an X-linked testis-specific gene, TEX11 expression is present in late-pachytene spermatocytes and in round and elongated spermatids (4), nevertheless, TEX11 staining can be seen in spermatogonia, spermatocytes and post-meiotic sperm in the seminiferous tubules in our study. The difference may arise from the antibody specificity and the different mutation site of TEX11. *TEX11* mutations have been identified in many patients with azoospermia (25). Yu et al. reported a deletion mutation in exon 3 in infertile patients with meiotic arrest, representing a 2.5% incidence (26). Yatsenko et al. identified three splicing mutations and two missense mutations in infertile men (4); Yang et al. verified one frameshift mutation of *TEX11* in two brothers with azoospermia, and heterozygous mutations were also found in his mother (6). Clinic-pathological variables of infertile patients with *TEX11* mutation in the published literatures and our study are shown in **Table 2**. The high frequency of *TEX11* mutations in men with azoospermia suggests a critical role in human spermatogenesis, and deficiency in *TEX11* causes meiotic arrest and male infertility. The abundance of *TEX11* mutations may be a great help in auxiliary analysis of male infertility, however, it also increases the difficulty of identifying causal mutations for male infertility.

**Table 2:** Mutations of TEX11 reported for azoospermia patients in published literature and our data.

Position	Nucleotide change	Protein/RNA change	Testicular sperm	Patients (n)	Reference
Exon 6	405C>T	Silent mutation, A135spl d^b^	Few sperm	1	(4)
Exon 7	466A>G	Missense mutation, M156V	No sperm	1	(4)
Exon 9-11	607del237bp	203del79aa	Few sperm	2	(4)
Intron 10	748+1G>A^c^	L249spl d^b^	No sperm	1	(4)
Intron 21	1793+1G>C^c^	R597spl d^b^	No sperm	1	(4)
Exon 24	2047G>A	Missense mutation, A683T	Few sperm	1	(4)
Exon 6	349T>A	Missense mutation, W117R	No sperm	1	(3)
Exon 6	405C>T	Silent mutation	No sperm	1	(3)
Exon 7	424G>A	Missense mutation, V142I	No sperm	1	(3)
Exon 7	515A>G	Missense mutation, Q172R	No sperm	1	(3)
Exon 10	731C>T	Missense mutation, T244I	No sperm	1	(3)
Exon 16	1258Ins (TT)	Frameshift mutation, 1258GATG→TTGGTA	No sperm	1	(3)
Exon 26	2243T>C	Missense mutation, V748A	No sperm	1	(3)
Exon 27	2319T>C	Silent mutation	No sperm	1	(3)
Intron 3	-17T>C^c^	Intronic alteration	No sperm	1	(3)
Intron 5	-48G>A^c^	Intronic alteration	No sperm	1	(3)
Intron 10	+42C>A^c^	Intronic alteration	No sperm	1	(3)
Intron 12	-28T>C^c^	Intronic alteration	No sperm	1	(3)
Intron 15	-64G>A^c^	Intronic alteration	No sperm	1	(3)
Intron 21	-1G>A^c^	Intronic alteration	No sperm	1	(3)
Intron 22	-37A>G^c^	Intronic alteration	No sperm	1	(3)
Intron 24	+119G>A^c^	Intronic alteration	No sperm	1	(3)
Intron 27	-55A>C^c^	Intronic alteration	No sperm	1	(3)
Intron 28	-44A>G^c^	Intronic alteration	No sperm	1	(3)
Exon 29	2568G>T	Missense mutation, W856C	No sperm	2	(25)
Exon 3	151_154del	D51 frame-shift mutation	No sperm	1	(26)
Intron 21	1796 + 2T > G	Splicing mutation, 599K spl d	No sperm	2	(15)
Intron 16	1426-1C > T	Splicing mutation, 476A spl d	No sperm	6	(15)
Exon 30	2613G > T	Missense mutation, W871C	No sperm	2	(15)
Exon 12	1051G > T	Nonsense mutation, E351*	No sperm	1	(15)
Exon 16	1254dupA	Frameshift mutation, N418K fs*10	No sperm	1	(15)
Exon 5	298delG	Frameshift mutation, V85L fs*5	No sperm	1	(15)
Exon 12	857delA	Frameshift mutation K286R fs*5	No sperm	1	(15)
Exon 26	2240C>A	Missense mutation p.S747X	No sperm	1	(27)
Exon 16	1337G>T	Missense mutation p.R446M	No sperm	1	(27)
Exon 16	1246C>T	Missense mutation p.Q416X	No sperm	1	(27)
Exon 5	313C>T	Nonsense mutation, R105* p.R105*	No sperm	1	This study
Exon 7	427A>C	Missense mutation, K143Q K143Q	No sperm	1	This study
Exon 29	2575G>A	Missense mutation, G859R G859R	No sperm	2	This study

^a^TEX11 mutations were mapped to isoform 2 (GenBank accession number, NM_031276); ^b^The term spl d represents the splicing donor sit; ^c^+1 refers to the first base of a given intron, and -1 denotes the last base. TEX11: testis expressed 11; del: deletion; bp: base pair; Ins: insertion.

**Figure 4:**
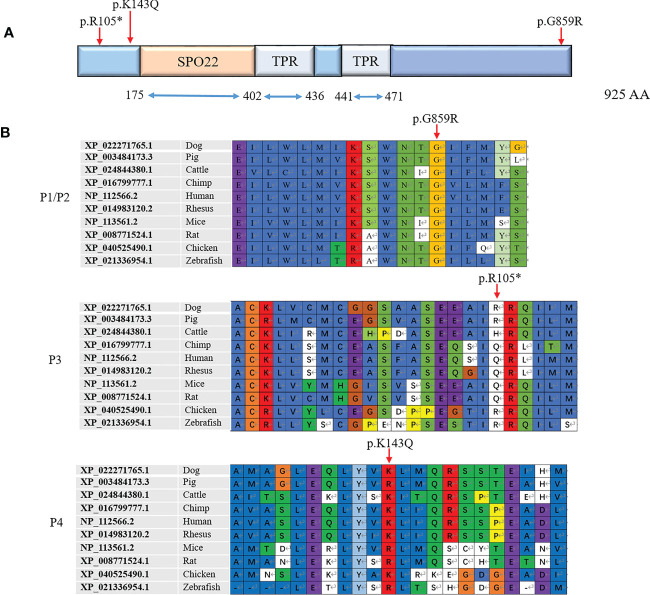
Three novel mutations identified in the TEX11 gene. **(A)** Predicted TEX11 domains with a SPO22 motif (amino acid positions 175-402) and multiple TPR-containing regions. The mutations found were shown under the predicted protein domains for TEX11. **(B)** Amino acid sequence alignment of TEX11 from different species. The red arrow indicates the mutated amino acid. TEX11, testis expressed 11; SPO22, sporulation domain; TPR, tetratricopepetide repeat; WT, wild type; AA, amino acid.

*TEX11* was reported to be an X-linked meiosis-specific gene, and contain a meiosis-specific sporulation (SPO22) domain (175-402AA) and repetitive tetratricopeptide repeat (TPR) domains (402-436AA and 441-471AA) (**Figure 4A**), which are commonly observed in scaffold proteins and exhibit a wide range of molecular recognition modes (28, 29). Extensive studies revealed that TEX11 plays an essential role in meiotic recombination, the repair of DNA double-strand breaks, meiotic crossover and chromosomal synapsis (6, 14, 30). Mutations in SPO22/ZIP4, which are the homologues of TEX11 in budding yeast and Arabidopsis, led to defects in meiosis (31, 32). Therefore, the function of TEX11 in meiosis is highly conserved from budding yeast to humans. Moreover, TPRs is composed of helix-turn-helix repeats that typically appear in tandem and pack with each other to form super-helical structures with various curvatures. In brief, TPRs are protein-protein interaction modules that can provide docking surfaces for other molecules (4). What’s more, some TPR proteins orchestrate different activities by integrating signals from multiple interacting partners (33). TEX11 was reported to contain repetitive TPR domains, which may provide docking surfaces for SYCP2 to form synaptonemal complex, and involved in chromosomal synapsis and crossover formation in meiosis. In the present study, we identified three novel *TEX11* mutations in patients and their mother. The *TEX11* p.R105* mutation displayed in our current study resulted in spermatogenic failure for loss of SPO22 and TPR domains; the other two missense mutations (p.G859R and p.K143Q) identified in our report were neither in SPO22 nor TPR domains; however, the Gly859 residue was found to be highly conserved across several species (**Figure 4B**) and histological analysis of testis biopsy obtained from the patient with Gly859 missense mutation showed meiotic arrest and no post-meiotic germ cells were observed in the seminiferous tubules. The testicular histology of two brothers carrying p.G859R mutation suggested that this mutation caused meiotic arrest. The mutations of p.G859R and p.K143Q are not in the known functional domains of TEX11, and how it affects meiosis is unclear. What is known is that TEX11 forms distinct foci on homologous chromosomes that synapse with each other, therefore these mutations may affect the tertiary structure, disrupting its function or stability. Further study will be necessary to clarify the molecular determinants that control TEX11 function and the connection between function domain and function.

In conclusion, the current report presents three pathogenic mutations in *TEX11* gene in four patients which are possibly associated with male infertility. All patients presented with azoospermia at reproductive age without any other manifestations. This study provides novel *TEX11* mutations in infertile men with meiotic arrest, which not only helps to ascertain the exact genetic cause in each patient but also facilitates the counseling of family members about their reproductive health. While we presented *TEX11* mutations in infertile men, causality of these variants has not been definitively proven.

## Data availability statement

The original contributions presented in the study are included in the article/**Supplementary Material**. Further inquiries can be directed to the corresponding authors.

## Author contributions

JS: Conceptualization, methodology, investigation, data curation. YS: Methodology, investigation, funding acquisition. XL: Data curation, writing-review. XuZ: Visualization and writing-review. XiZ: Methodology, writing-review, and editing. All authors contributed to the article and approved the submitted version.

## References

[1] Mueller JL Mahadevaiah SK Park PJ Warburton PE Page DC Turner JM. The mouse X chromosome is enriched for multicopy testis genes showing postmeiotic expression. Nat Genet (2008) 40 6:794–9. doi: 10.1038/ng.126 PMC2740655 18454149

[2] Zheng K Yang F Wang PJ. Regulation of male fertility by X-linked genes. J Androl (2010) 31 1:79–85. doi: 10.2164/jandrol.109.008193 19875494 PMC2931805

[3] Yang F Silber S Leu NA Oates RD Marszalek JD Skaletsky H. TEX11 is mutated in infertile men with azoospermia and regulates genome-wide recombination rates in mouse. EMBO Mol Med (2015) 7 9:1198–210. doi: 10.15252/emmm.201404967 PMC4568952 26136358

[4] Yatsenko AN Georgiadis AP Röpke A Berman AJ Jaffe T Olszewska M. X-Linked TEX11 mutations, meiotic arrest, and azoospermia in infertile men. N Engl J Med (2015) 372 22:2097–107. doi: 10.1056/NEJMoa1406192 PMC4470617 25970010

[5] Xie C Chen X Liu Y Wu Z Ping P. Multicenter study of genetic abnormalities associated with severe oligospermia and non-obstructive azoospermia. J Int Med Res (2018) 46 1:107–14. doi: 10.1177/0300060517718771 PMC6011285 28730893

[6] Yang F Gell K van der Heijden GW Eckardt S Leu NA Page DC. Meiotic failure in male mice lacking an X-linked factor. Genes Dev (2008) 22 5:682–91. doi: 10.1101/gad.1613608 PMC2259036 18316482

[7] Choi Y Jeon S Choi M Lee MH Park M Lee DR. Mutations in SOHLH1 gene associate with nonobstructive azoospermia. Hum Mutat (2010) 31 7:788–93. doi: 10.1002/humu.21264 20506135

[8] Miyamoto T Hasuike S Yogev L Maduro MR Ishikawa M Westphal H. Azoospermia in patients heterozygous for a mutation in SYCP3. Lancet (2003) 362 9397:1714–9. doi: 10.1016/S0140-6736(03)14845-3 14643120

[9] Ropke A Tewes AC Gromoll J Kliesch S Wieacker P Tuttelmann F. Comprehensive sequence analysis of the NR5A1 gene encoding steroidogenic factor 1 in a large group of infertile males. Eur J Hum Genet (2013) 21 9:1012–5. doi: 10.1038/ejhg.2012.290 PMC3746266 23299922

[10] Imken L Rouba H El Houate B Louanjli N Barakat A Chafik A. Mutations in the protamine locus: association with spermatogenic failure? Mol Hum Reprod (2009) 15 11:733–8. doi: 10.1093/molehr/gap056 19602509

[11] Mou L Wang Y Li H Huang Y Jiang T Huang W. A dominant-negative mutation of HSF2 associated with idiopathic azoospermia. Hum Genet (2013) 132 2:159–65. doi: 10.1007/s00439-012-1234-7 23064888

[12] Hu Z Xia Y Guo X Dai J Li H Hu H. A genome-wide association study in Chinese men identifies three risk loci for non-obstructive azoospermia. Nat Genet (2011) 44 2:183–6. doi: 10.1038/ng.1040 22197933

[13] Hu Z Li Z Yu J Tong C Lin Y Guo X. Association analysis identifies new risk loci for non-obstructive azoospermia in Chinese men. Nat Commun (2014) 5:3857. doi: 10.1038/ncomms4857 24852083

[14] Adelman CA Petrini JH. ZIP4H (TEX11) deficiency in the mouse impairs meiotic double strand break repair and the regulation of crossing over. PloS Genet (2008) 4 3:e1000042. doi: 10.1371/journal.pgen.1000042 18369460 PMC2267488

[15] Ji Z Yao C Yang C Huang C Zhao L Han X. Novel hemizygous mutations of TEX11 cause meiotic arrest and non-obstructive azoospermia in Chinese han population. Front Genet (2021) 12:741355. doi: 10.3389/fgene.2021.741355 34621296 PMC8491544

[16] Tan YQ Tu C Meng L Yuan S Sjaarda C Luo A. Loss-of-function mutations in TDRD7 lead to a rare novel syndrome combining congenital cataract and nonobstructive azoospermia in humans. Genet Med (2019) 21 5:1209–17. doi: 10.1038/gim.2017.130 31048812

[17] Cooke HJ Saunders PT. Mouse models of male infertility. Nat Rev Genet (2002) 3 10:790–801. doi: 10.1038/nrg911 12360237

[18] Tamowski S Aston KI Carrell DT. The use of transgenic mouse models in the study of male infertility. Syst Biol Reprod Med (2010) 56 3:260–73. doi: 10.3109/19396368.2010.485244 20536325

[19] Eggers S DeBoer KD van den Bergen J Gordon L White SJ Jamsai D. Copy number variation associated with meiotic arrest in idiopathic male infertility. Fertil Steril (2015) 103 1:214–9. doi: 10.1016/j.fertnstert.2014.09.030 25439847

[20] Rohozinski J Bishop CE. The mouse juvenile spermatogonial depletion (jsd) phenotype is due to a mutation in the X-derived retrogene, mUtp14b. Proc Natl Acad Sci U.S.A. (2004) 101 32:11695–700. doi: 10.1073/pnas.0401130101 PMC511039 15289605

[21] Danshina PV Geyer CB Dai Q Goulding EH Willis WD Kitto GB. Phosphoglycerate kinase 2 (PGK2) is essential for sperm function and male fertility in mice. Biol Reprod (2010) 82 1:136–45. doi: 10.1095/biolreprod.109.079699 PMC2802118 19759366

[22] Avasthi P Scheel JF Ying G Frederick JM Baehr W Wolfrum U. Germline deletion of Cetn1 causes infertility in male mice. J Cell Sci (2013) 126(Pt 14):3204–13. doi: 10.1242/jcs.128587 PMC3711207 23641067

[23] Jiang L Li T Zhang X Zhang B Yu C Li Y. RPL10L is required for Male meiotic division by compensating for RPL10 during meiotic sex chromosome inactivation in mice. Curr Biol (2017) 27 10:1498-1505.e6. doi: 10.1016/j.cub.2017.04.017 28502657

[24] Tardif S Akrofi AS Dass B Hardy DM MacDonald CC. Infertility with impaired zona pellucida adhesion of spermatozoa from mice lacking TauCstF-64. Biol Reprod (2010) 83 3:464–72. doi: 10.1095/biolreprod.109.083238 PMC2924806 20463354

[25] Sha Y Zheng L Ji Z Mei L Ding L Lin S. A novel TEX11 mutation induces azoospermia: a case report of infertile brothers and literature review. BMC Med Genet (2018) 19 1:63. doi: 10.1186/s12881-018-0570-4 29661171 PMC5902858

[26] Yu XC Li MJ Cai FF Yang SJ Liu HB Zhang HB. A new TEX11 mutation causes azoospermia and testicular meiotic arrest. Asian J Androl (2021) 23 5:510–5. doi: 10.4103/aja.aja_8_21 PMC8451497 33762476

[27] An M Liu Y Zhang M Hu K Jin Y Xu S. Targeted next-generation sequencing panel screening of 688 Chinese patients with non-obstructive azoospremia. J Assost Reprod Genet (2021) 38(8):1997–2005. 10.1007/s10815-021-02154-9 PMC8417191 33728612

[28] D'Andrea LD Regan L. TPR proteins: the versatile helix. Trends Biochem Sci (2003) 28 12:655–62. doi: 10.1016/j.tibs.2003.10.007 14659697

[29] Perez-Riba A Itzhaki LS. The tetratricopeptide-repeat motif is a versatile platform that enables diverse modes of molecular recognition. Curr Opin Struct Biol (2019) 54:43–9. doi: 10.1016/j.sbi.2018.12.004 30708253

[30] Dapper AL Payseur BA. Molecular evolution of the meiotic recombination pathway in mammals. Evolution (2019) 73 12:2368–89. doi: 10.1111/evo.13850 PMC7050330 31579931

[31] Chelysheva L Gendrot G Vezon D Doutriaux MP Mercier R Grelon M. Zip4/Spo22 is required for class I CO formation but not for synapsis completion in arabidopsis thaliana. PloS Genet (2007) 3 5:e83. doi: 10.1371/journal.pgen.0030083 17530928 PMC1877879

[32] Tsubouchi T Zhao H Roeder GS. The meiosis-specific zip4 protein regulates crossover distribution by promoting synaptonemal complex formation together with zip2. Dev Cell (2006) 10 6:809–19. doi: 10.1016/j.devcel.2006.04.003 16740482

[33] Pyatnitskaya A Andreani J Guerois R De Muyt A Borde V. The Zip4 protein directly couples meiotic crossover formation to synaptonemal complex assembly. Genes Dev (2022) 36 1-2:53–69. doi: 10.1101/gad.348973.121 34969823 PMC8763056

