# Supplementary material for: Novel mutations of TEX11 are associated with non-obstructive azoospermia
Source: Front Endocrinol (Lausanne). 2023 Apr 14;14:1159723. doi: 10.3389/fendo.2023.1159723 (PMC10140331; doi:10.3389/fendo.2023.1159723)
Supplement: Supplementary file 1 [file Table_1.docx]

Supplementary information：


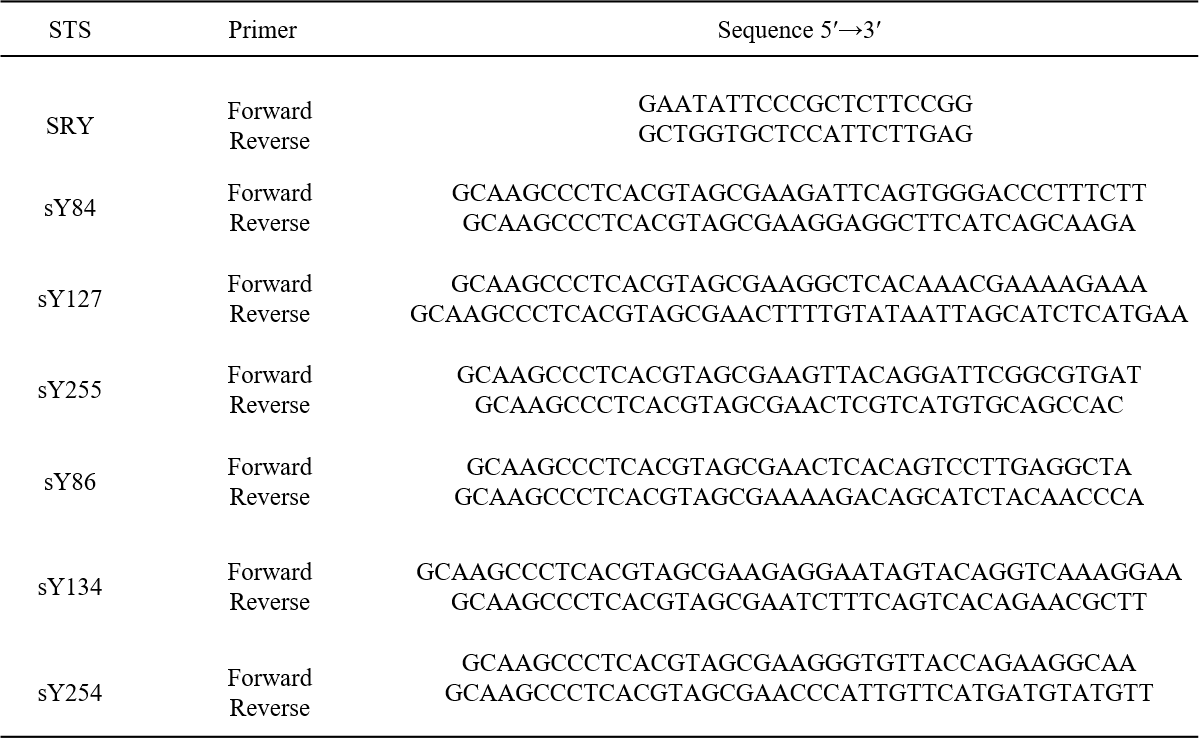
Table 1. STS and gene-specific primer sequences for Y chromosome microdeletion analysis

STS, sequence-tagged site

Table 2. Primer sequences used for TEX11 mutation

| Mutation | Primer | Sequence 5′→3′ |
| --- | --- | --- |
|  |  |  |
| c.G2575A | Forward  Reverse | GGAGCACAGACCTTCCTAAA  TCCCTCCTTACCTGAGTTTC |
|  |  |  |
| c.C313T | Forward  Reverse | CCGGCTAAAACGGTGAAAC  GTAAGCGGCAGACCTAACCT |
|  |  |  |
| c.A427C | Forward  Reverse | AGAATGGTTGGATGCTGGAA  TGATGGCAAAAGGAGGAAAC |
|  |  |  |
